# Band Gap Engineering of Multi-Junction Solar Cells: Effects of Series Resistances and Solar Concentration

**DOI:** 10.1038/s41598-017-01854-6

**Published:** 2017-05-11

**Authors:** Joya Zeitouny, Eugene A. Katz, Alain Dollet, Alexis Vossier

**Affiliations:** 1 CNRS-PROMES, 7 Rue du Four Solaire, 66120 Odeillo, France; 2 Department of Solar Energy and Environmental Physics, Jacob Blaustein Institutes for Desert Research, Ben-Gurion University of the Negev, Sede Boqer Campus, 84990 Israel

## Abstract

Multi-junction (MJ) solar cells are one of the most promising technologies achieving high sunlight to electricity conversion efficiency. Resistive losses constitute one of the main underlying mechanisms limiting their efficiency under high illumination. In this paper, we study, by numerical modeling, the extent to which a fine-tuning of the different electronic gaps involved in MJ stacks may mitigate the detrimental effects of series resistance losses for concentration-dependent and independent series resistances. Our results demonstrate that appropriate bandgap engineering may lead to significantly higher conversion efficiency at illumination levels above ~1000 suns and series resistance values typically exceeding 0.02 Ω cm^2^, due to lower operating current and, in turn, series resistance losses. The implications for future generations of solar cells aiming at an improved conversion of the solar spectrum are also addressed.

## Introduction

Ultra-high power conversion efficiency (PCE) can be achieved by the combination of (1) advanced solar cell architecture allowing an efficient use of the broad solar energy spectrum and (2) optical concentration, toward reducing the fundamental asymmetry between absorbed and emitted radiation, and thus the associated Boltzmann losses^1^. To date, record conversion efficiency of 46% has been achieved on quadruple-junction solar cell at an illumination level of 508 suns^2^ (1 sun = 100 mW/cm^2^). The path toward 50% efficiency and above will undoubtedly require even more sophisticated cell architectures, as well as higher illumination levels. One of the fundamental mechanisms limiting the cell efficiency under high illumination includes the series resistance losses, stemming from the finite electrical conductivity of the materials involved^3^. Series resistance losses are known to affect the cell efficiency through a drop in the Fill-Factor (FF), which amplitude is a function of the cell architecture used, the materials involved, and the illumination level to which the cell is exposed. Various strategies have been proposed in order to mitigate the effects of series resistance losses. In particular, appropriate tailoring of the front grid design should be realized toward finding the optimal balance between grid and contact resistance, emitter sheet resistance, and shading associated with the front-contact grid^4, 5^. Decreasing the cell dimensions can also lead to decreased series resistance losses, through a drop in the area-related contribution of the series resistance^3, 6^. However, these strategies only allow a moderate improvement in the cell efficiency and the peak illumination level for which the maximum efficiency is achieved does not exceed 1000 suns^7–9^. Vertical solar cells were proposed in the mid 70’s as a way to dramatically reduce the effects of series resistance, by decoupling propagation of light and diffusion of charge carriers in the cell^10, 11^. The concept was later extended to Multi-Junction (MJ) solar cells by Braun *et al*., who theoretically demonstrated that such cell architectures could reach ultra-high efficiencies at illumination levels as high as 10,000 suns, without any significant alteration due to series resistance effects^12^. However, this concept remains theoretical and its practical implementation will necessarily require overcoming numerous technological challenges.

MJ cells involve a combination of semiconductor materials with different bandgaps in order to better absorb the solar spectrum. They are usually designed assuming no series resistance losses in the cell. The optimal combination of electronic gaps leading to the highest PCE efficiency is derived from iso-efficiency curves which provide for manufacturers a certain degree of freedom in the choice of the materials involved in the MJ stack. However, supplemental technological constraints (such as the lattice matching between each subcell which is usually required to minimize the amount of threading dislocations between the different layers) may constrain significantly the possible choice of materials.

It was recently pointed out that the amplitude with which series resistance affects the cell efficiency largely depends on the semiconductor bandgap^13^. Series resistance losses appear to be higher for low bandgap materials. Indeed, at fixed illumination intensity, higher photocurrent is generated in low bandgap materials due to their capacity to absorb light over an extended range of wavelengths. Accordingly, the deterioration of their electrical characteristics is stronger in comparison to high bandgap materials.

Thus, series resistance losses could alter the optimal combination of bandgaps leading to the highest achievable efficiency, especially at high illumination levels. If so, one should consider series resistance losses when designing MJ device operating at ultra-high concentration. To the best of our knowledge, the following question remains open: Is it possible to increase PCE of MJ cells at high illumination level by carefully tailoring bandgap combination in subcells toward minimizing resistive losses?

In this study, we use numerical modeling to investigate: (1) how series resistances can affect the performance of concentrator multi-junction solar cells and (2) how this parameter can alter the optimal bandgap combination of the different junctions in the cell.

## Methodology

The maximum PCE attainable with a wide variety of solar cells can be derived using the detailed balance formalism, originally suggested by William Shockley and Hans Queisser in 1961^14^, and which has been largely described elsewhere^15^.

The “ideal” current-voltage characteristics for a one-diode model (accurate enough to describe the behavior of solar cells under high illumination levels^16^) and in the absence of any series resistance losses, can be deduced from the above formalism, leading to the well-known equation:

1 $$I={I}_{ph}-{I}_{0}(\exp (\frac{qV}{nkT})-1)$$

where *I*_*ph*_ and *I*_*0*_ are the photo-generated and the dark currents (equations (2) and (3) respectively), *n* is the ideality factor, *q*, *k* and *T* are the elementary charge, the Boltzmann constant, and the ambient temperature respectively, and *V* is the voltage. This ideal model assumes radiative recombination as the sole recombination mechanism occurring in the cell, thus placing an upper bound on the maximum voltage achievable.

2 $${I}_{ph}=q\times {\int }_{{E}_{\min }}^{{E}_{\max }}f(E)dE$$

3 $${I}_{0}=\frac{q}{k}\times \frac{15\sigma }{{\pi }^{4}}\times {T}^{3}\times {\int }_{u}^{\infty }\frac{{x}^{2}}{{e}^{x}-1}dx$$

with *f(E)* the spectral energy distribution of AM1.5D, *E*_*min*_ and *E*_*max*_ the minimum and maximum energy of the photons absorbed by the solar cell, *σ* the Stephan-Boltzmann constant and $u=\frac{{E}_{g}}{kT}$.

Operating PV cells under concentrated sunlight necessarily leads to non-negligible series-resistance losses as a result of the high photo-generated current, thus requiring a modification of the previous equation:

4 $$I={I}_{ph}-{I}_{0}(\exp (\frac{qV+I{R}_{s}}{nkT})-1)$$

where *R*_*s*_ is the series resistance (shunt resistance is assumed to be infinite).

In the case of current-matched MJ solar cells, the current-voltage characteristics simply correspond to the sum of each individual subcell IV curve (which is also described by eq. 4), taking into account the current constraint imposed by the series connection between each *pn* junction. Because of the bandgap dependence of *R*_*s*_-losses, the extent to which any particular MJ stack is affected by series resistance losses is likely to vary significantly depending on the typical values of the electronic gaps involved. The optimal combination of electronic gaps leading to the highest conversion efficiency was investigated over a broad range of series resistances and concentrations, using a genetic algorithm. This method, inspired by natural selection, is a powerful tool used for optimization^17^. It uses a set of electronic gaps (whose number depends on the cell architecture investigated) as an initial population, and various genetic operations (namely (mutations, cross-over, breeding) applied toward minimizing an objective function, defined here as 1-η (η being the photovoltaic conversion efficiency). The optimal set of electronic gaps for any particular combination of series resistance and concentration is thus defined as the outcome of successive generations selected for their ability to minimize the objective function.

### Series resistance scenario

It is commonly assumed in the literature that the “lumped” series resistance value is “light-intensity” independent^6^. Previous research showed that the illumination level to which the cells are submitted should be taken into account when designing concentrator solar cells: in such case, the “lumped” series resistance value was shown to be strongly correlated to the illumination level and its value appears to decrease significantly with increasing sunlight concentration^3^. In this study, both scenarios were investigated and compared, namely (1) an illumination-independent *R*_*s*_ value, whose value is selected for being representative of the state-of-the-art concentrator solar cells (typically less or equal to 0.01 ohm cm^2^ for triple^18^ or even quadruple-junction^19^ solar cells, even if values comprised between 0.01 and 0.025 ohm cm^2^ are also reported in the literature, depending on the cell area, and the grid pitch design and dimensions^18^) (2) an illumination-dependent *R*_*s*_ value, estimated using the procedure described in ref. 3, and assuming the lumped-*R*_*s*_ value at any particular illumination level is the sum of two distinct contributions: a variable term associated with series resistance components likely to be optimized depending on the illumination level to which the cell is submitted (emitter, front contact, grid…) and a fixed term associated with illumination-independent contributions (vertical resistance, tunnel diodes…). For most direct bandgap III-V materials commonly used in concentrator solar cells, the “fixed” resistance contribution associated with the vertical flow of current is usually negligible relative to the concentration-dependent term up to ~3000 suns, an illumination level above which the “fixed” and the “concentration-dependent” components typically reach comparable values^3^ (even though Ge-based triple junction solar cells may show different trends under very high illumination levels). Typical values for the “concentration-dependent” resistance considered in this work were retrieved from ref. 3, with a lumped series resistance value assumed to vary logarithmically between 0.46 Ω cm^2^ at one sun illumination, and 0.15 mΩ cm^2^ at 10000 suns. Additional 0.14 mΩ cm^2^ tunnel junction resistances^20^ were added in the concentration-dependent *R*_*s*_ model to account for the presence of tunnel diodes between each subcell.

## Results

To better assess the amplitude of the series resistance effect as well as the improvement in the cell efficiency attainable with a MJ stack designed to minimize *R*_*s*_-losses, we plotted iso-efficiency curves for several MJ cell architectures, for a broad range of illumination intensities and series resistance values. Iso-efficiency curves are derived after solving eq. 2 for the different subcells using Matlab R2013a environment (Mathworks Inc, Natick, MA, USA). They represent the efficiency with which solar energy is converted into electricity as a function of the bandgap of the different semiconductor materials in the MJ stack. This approach allows calculating the optimal bandgap combination and the maximum efficiency of the MJ cell.

**Figure 1:**
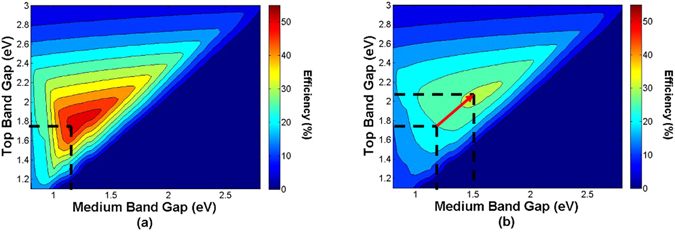
Iso-efficiency curves of 3-J cells under 2500 suns for different lumped series resistance values: (**a**) 0.01 Ω cm^2^ and (**b**) 0.05 Ω cm^2^.

Figure 1 shows the iso-efficiency curves of triple junction (3-J) cells under a concentration of 2500 suns for two different values of total series resistances in the illumination-independent *R*_*s*_ scenario: (a) 0.01 Ω cm^2^ and (b) 0.05 Ω cm^2^, and assuming a 0.7 eV bottom cell (corresponding to a Ge subcell, which has been widely used as bottom cell material in triple-junction cells).

Beyond the significant decrease in the maximum efficiency attainable when increasing the total series resistance value from 0.01 to 0.05 Ω cm^2^, a strong shift in the optimal electronic gap combination can be noticed: while 0.01 Ω cm^2^ triple-junction solar cell structures show a maximum efficiency for a combination of 1.75/1.18/0.7 eV semiconductor materials, cells characterized by a 0.05 Ω cm^2^ series resistance value demonstrate a major displacement in their optimal bandgap combination toward higher bandgap values: the maximum efficiency is achieved for a 2.07/1.58/0.7 eV cell architecture.

In the illumination-dependent *R*_*s*_ scenario, one can observe very different trends: because of the reduced penalty associated with series resistance losses at high illumination levels, both the decrease in the maximum efficiency attainable and the shift in the optimal combination of electronic gaps appear to be much less pronounced.

**Figure 2:**
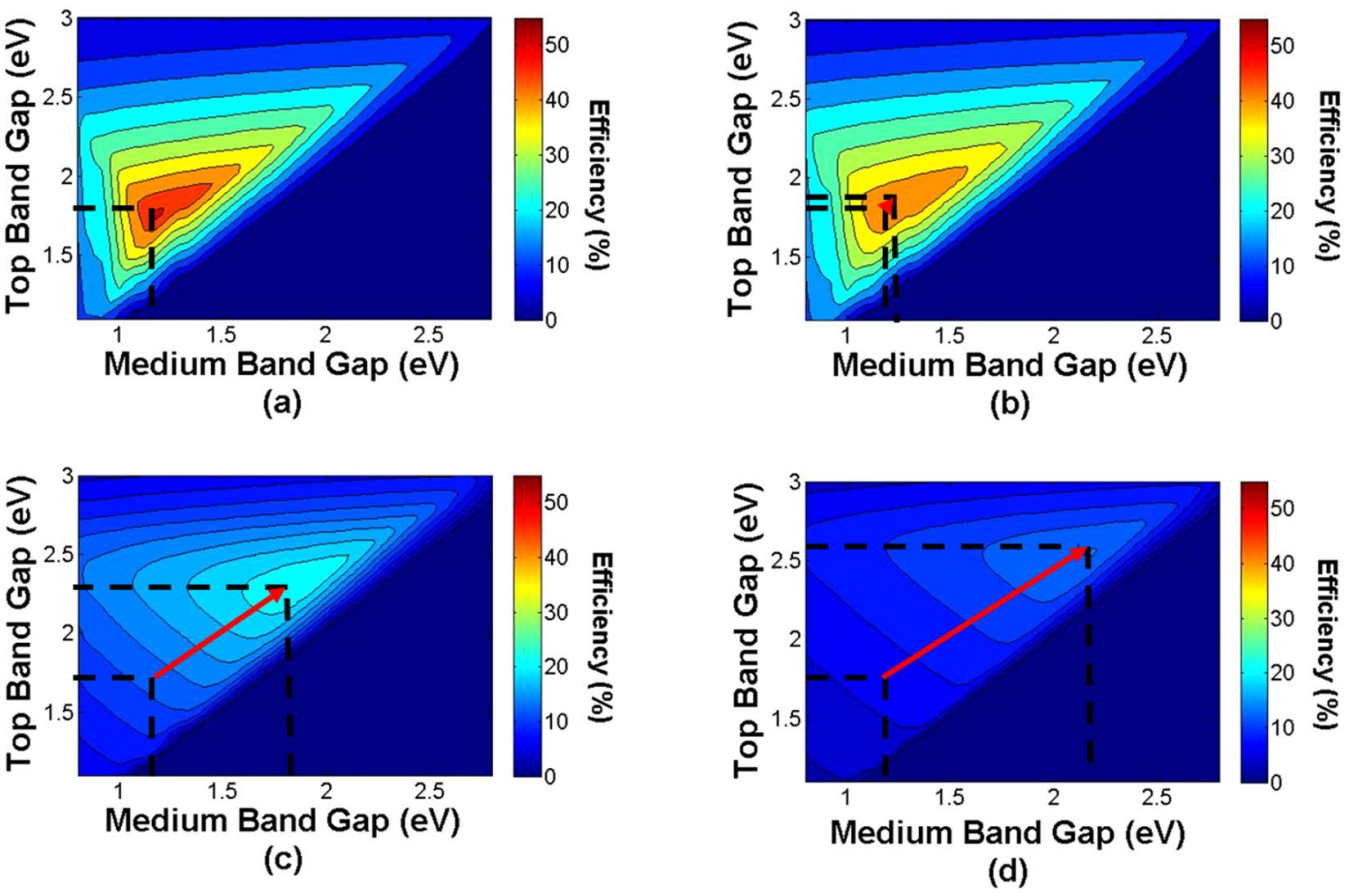
Iso-efficiency curves of 3-J cells with a total series resistance of 0.05 Ω cm^2^ and for illumination levels of (**a**) 1 sun (**b**) 1000 suns (**c**) 5000 suns and (**d**) 10,000 suns.

Figure 2 illustrates the iso-efficiency curves of a 3-J cell under different concentrations: (a) 1 sun, (b) 1,000 suns, (c) 5,000 suns and (d) 10,000 suns and with a total series resistance of 0.05 Ω cm^2^ also assuming a 0.7 eV bottom cell.

The effects of the illumination level on the optimal bandgap combination in Fig. 2 appear to be relatively modest up to illumination level equivalent to 1,000 suns. At higher intensities, however, we observe a noticeable change in the optimal bandgap combination toward higher *E*_*g*_ values. These results suggest that minimizing resistive losses in MJ cells should include a tailoring of the different electronic gaps.

In the illumination-dependent *R*_*s*_ scenario, the trends are rather different: due to the lower magnitude of the *R*_*s*_ losses at high illumination levels (because of the decreased *R*_*s*_ stemming basically from the front-contact grid and emitter optimization) the benefit for bandgap-optimization is almost negligible.

Table 1 summarizes the optimal band gap combinations for the reference case (optimal bandgap combination of 1.75/1.18/0.7 eV for no series resistance, at an illumination of 1 sun^21^) and for two series resistances values corresponding to a low *R*_*s*_ scenario (0.01 Ω cm^2^) and a high *R*_*s*_ scenario (0.05 Ω cm^2^) in the illumination-independent *R*_*s*_ case, for a broad range of illumination levels comprised between 1 and 10,000 suns. If the optimal bandgap combination remains unchanged up to illumination levels of 1000 suns (high series resistance scenario) or 2500 suns (low series resistance scenario), a significant change in the optimal bandgap combination can be observed at high illumination intensities. The amplitude of the shift depends on both the series resistance value and the illumination level to which the cell is submitted. In the most extreme illumination levels, the optimal bandgap combinations do not involve any low-bandgap material: because of the high resistive losses intrinsically associated with low *E*_*g*_ materials, the optimization procedure leads to an optimal combination of bandgaps involving only intermediate to high bandgap materials, leading to an increased voltage and a lowered current.

**Table 1:** Optimal combination of bandgaps of a 3-J cell architecture, for illumination levels comprised between 1 and 10,000 suns, and for a total series resistance value of 0, 0.01 and 0.05 Ω cm^2^.

Rs (ohm cm^2^)	X (suns)	E_g1_/E_g2_/E_g3_ (eV)
0 (Ref)	1	1.75/1.18/0.7
0.01	1	1.75/1.18/0.7
	1000	1.75/1.18/0.7
	2500	1.75/1.18/0.7
	5000	1.88/1.37/0.95
	10000	2.06/1.55/1.17
0.05	1	1.75/1.18/0.7
	1000	1.94/1.39/0.95
	2500	2.18/1.69/1.34
	5000	2.31/1.9/1.59
	10000	2.50/2.12/1.85

## Discussion

Table 2 summarizes the main electrical parameters derived for both cell architectures, at illumination levels of 1000, 2500, 5,000 and 10000 suns. A noticeable improvement in the photovoltaic performance of the optimized cell can be observed, mainly arising from the increased *FF* and *V*_*oc*_, relative to the non-optimized case. The increase in both *FF* and *V*_*oc*_ appears to be particularly strong for high *R*_*s*_ and/or under high illumination intensities. Proper tailoring of MJ cell architecture toward achieving high *PCE* under high illumination levels (1000 suns and above) should thus favor an increased *V*_*oc*_ and lower *I*_*sc*_ (as a consequence of the higher bandgaps involved in optimized MJ architectures), which, in turn, results in significant increase in *FF* and PCE values, since the voltage increase will compensate the decrease in the current value.

**Table 2:** Main photovoltaic parameters for 3-J cells with non-optimized and optimized bandgap combinations.

Series Resistances (ohm cm^2^)	Illumination	Non-optimized cell				Optimized cell			
		Fill Factor	V_oc_ (V)	J_sc_ (mA/cm^2^)	Efficiency (%)	Fill Factor	V_oc_ (V)	J_sc_ (mA/cm^2^)	Efficiency* (%)
0.0 (Ref)	1	—	—	—	—	0.89	2.84	18.18	51.07
0.01	1000	0.86	3.37	18.18 × 10^3^	58.18	0.86	3.37	18.18 × 10^3^	58.18 **(**+***0%*** **)**
	2500	0.78	3.44	4.54 × 10^4^	54.21	0.78	3.44	4.54 × 10^4^	54.21 **(**+***0%*** **)**
	5000	0.66	3.49	9.09 × 10^4^	46.54	0.74	4.03	7.5 × 10^4^	49.6 **(**+***3.06%*** **)**
	10000	0.45	3.55	18.18 × 10^4^	32.04	0.71	4.63	1.12 × 10^5^	40.54 **(**+***8.5%*** **)**
0.05	1000	0.65	3.37	18.18 × 10^3^	44.15	0.77	3.98	1.36 × 10^4^	46.31 **(**+***2.16%*** **)**
	2500	0.36	3.44	4.54 × 10^4^	24.87	0.72	4.91	2.28 × 10^4^	35.91 **(**+***11.04%*** **)**
	5000	0.25	3.49	6.79 × 10^4^	13.29	0.63	5.52	3.59 × 10^4^	27.54 **(**+***14.25%*** **)**
	10000	0.25	3.55	7.03 × 10^4^	6.91	0.57	6.2	4.90 × 10^4^	19.10 **(**+***12.19%*** **)**

*In parentheses: absolute efficiency gain relative to the non-optimized case.

**Figure 3:**
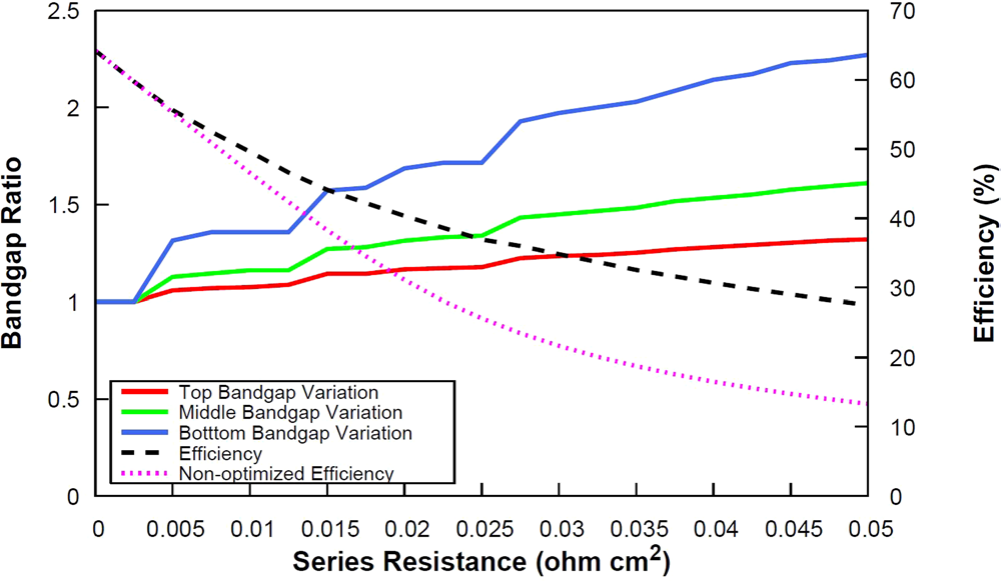
Ratio of optimized and non-optimized electronic gaps for a triple-junction solar cell (red line: top bandgap – green line: middle bandgap – blue line: bottom bandgap) and corresponding efficiency (purple dotted line: non-optimized cell – black dashed line: optimized cell), and as a function of the series resistance value, assuming an illumination of 5000 suns.

Figure 3 depicts the ratio between the optimized and non-optimized bandgaps for a triple junction solar cell together with their associated conversion efficiency, as a function of the lumped series resistance value, and assuming a 5000 suns illumination level. The shift in both the optimized bandgaps and the associated conversion efficiency, relative to the “non-optimized” reference architecture, appears to grow steadily with increasing lumped series resistance value: if the benefit for bandgap optimization is modest for 0.01 Ω cm^2^ cells (typical state-of-the-art concentrator solar cell) with an absolute gain in efficiency of ~3%, we observe a strong improvement in the bandgap-optimized cell relative to the reference architecture for a series resistance value of 0.05 Ω cm^2^, with an absolute efficiency gain of ~15%.

**Figure 4:**
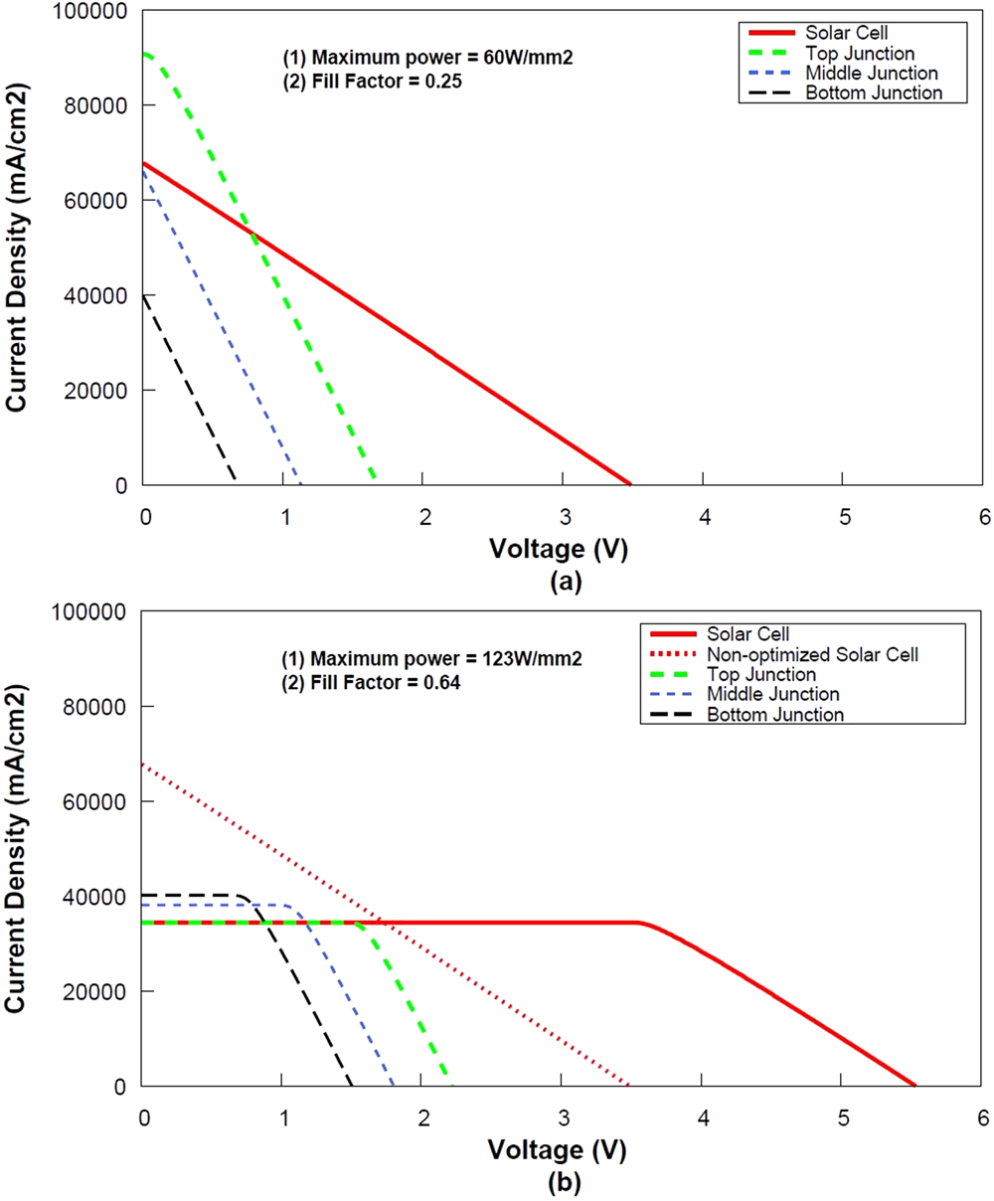
*I*-*V* curves of 3-J cells of non-optimized (1.75/1.18/0.7 eV) and optimized bandgap combination (Table 1) for total series resistance of 0.05 Ω cm^2^ operated under 5000 suns.

Figure 4 shows *I*-*V* curves for the 3 J cells operated under 5000 suns for a fixed series resistance scenario of 0.05 Ω cm^2^. Each graph includes different curves, showing the global electrical response of MJ cell (red curve) as well as the electrical response of each individual subcell (green, blue and black dashed curves) for a non-optimized (a) and optimized bandgap combination (b). The so-called “non-optimized *I-V* curves” correspond to the best combination for an ideal solar cell with zero series resistance under 1 sun intensity, namely 1.75/1.18/0.7 eV (top/middle/bottom junction). The bandgap combinations for the optimized cases (i.e the ones leading to the highest conversion efficiency, accounting for the series resistance as well as the concentration ratio) were taken from Table 1.

**Figure 5:**
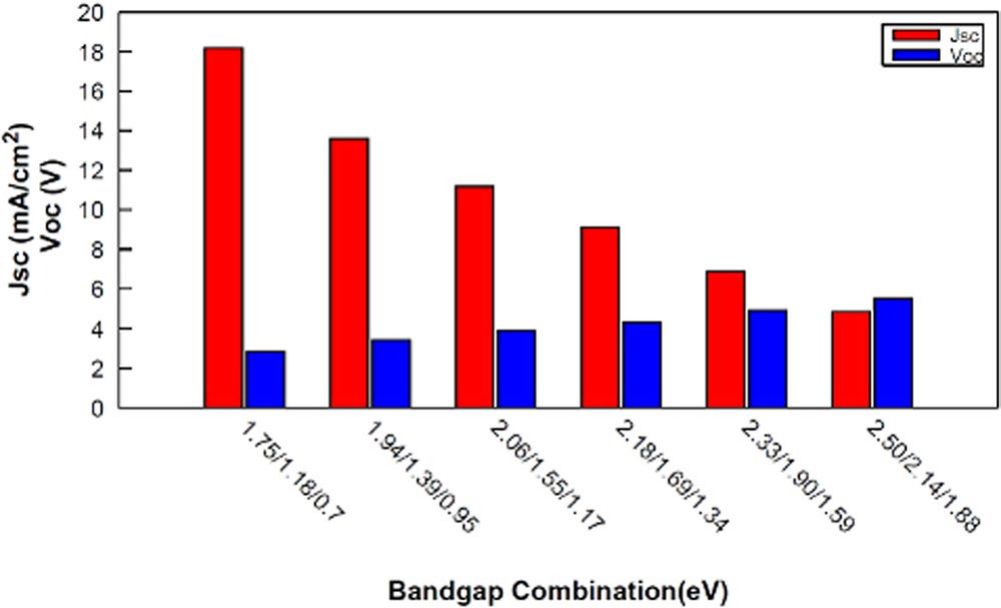
Evolution of J_sc_ and V_oc_ for different combination of bandgaps (Table 1) under an illumination of 1 sun.

Figure 5 represents the variation of the short-circuit current density and the open-circuit voltage under 1 sun illumination, and for different combination of electronic gaps taken from Table 1. As the values of the electronic gaps involved in the stack increase, we observe a significant drop in the short-circuit current density, together with an increase in the open-circuit voltage: under high illumination levels, the balance between *I*_*sc*_ and *V*_*oc*_ is thus drastically changed relative to the reference case (i.e no series resistance losses), to diminish the detrimental effects of *R*_*s*_ losses and increase the conversion efficiency.

**Figure 6:**
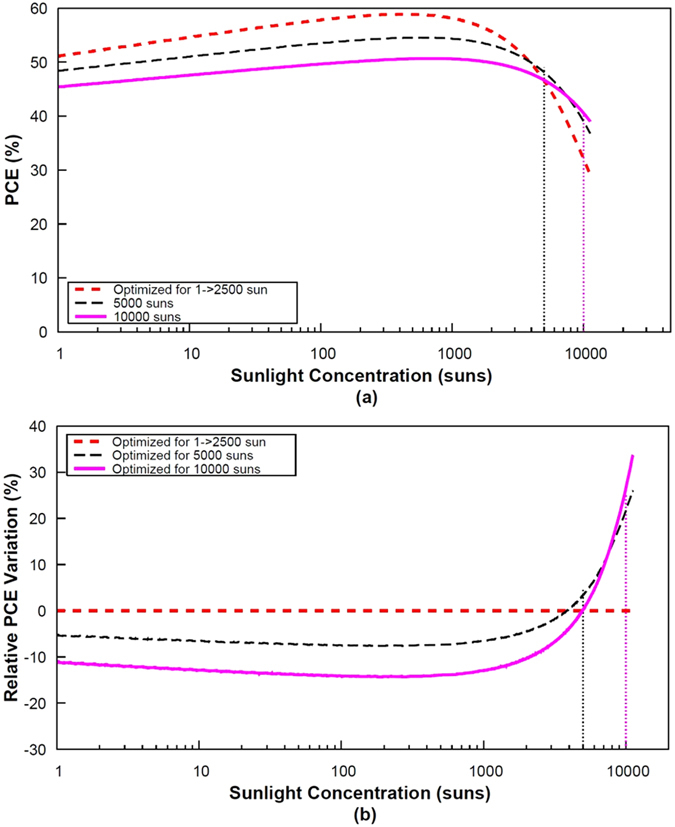
PCE (**a**) and relative PCE variation (**b**) as a function of sunlight concentration for 3-J cells with a total series resistance of 0.01 Ω cm^2^.

**Figure 7:**
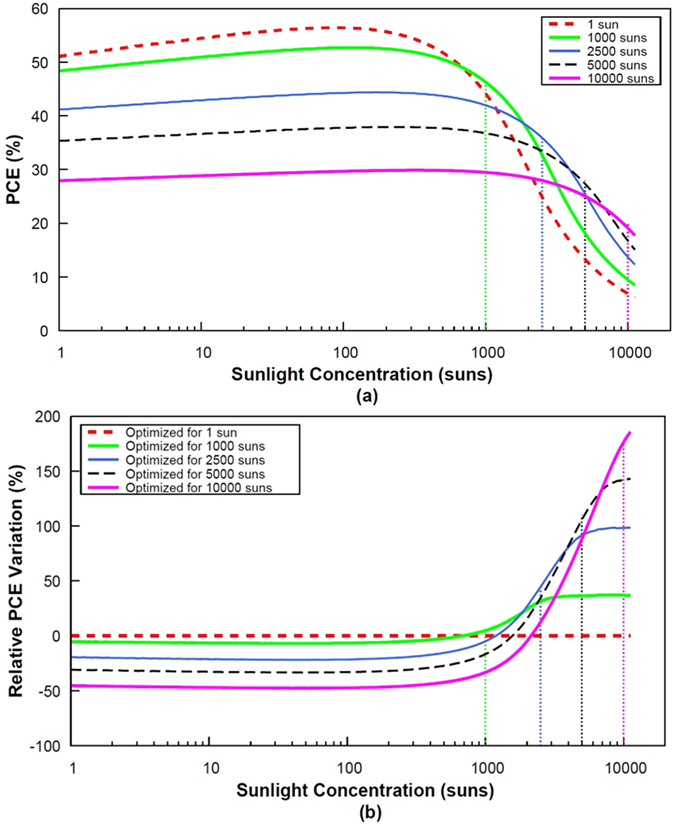
PCE (**a**) and relative PCE variation (**b**) as a function of sunlight concentration for 3-J cells with a total series resistance of 0.05 Ω cm^2^.

Fine-tuning of the bandgaps in the stack could thus potentially mitigate the effects of series resistance losses at high illumination levels. To better grasp the benefit of this approach, we represent the efficiency-concentration curves for a set of 3-J architectures whose bandgap combinations have been optimized for various illumination levels (from 1 to 10,000 suns) assuming a total series resistance of 0.01 Ω cm^2^ (Fig. 6) and 0.05 Ω cm^2^ (Fig. 7). For low series resistances (Fig. 6), the curves plotted for architectures optimized in the range 1–2500 suns are similar since series resistance losses remain insignificant and neither affect the conversion efficiency nor the bandgap combinations. For improved clarity, we determined the relative efficiency variation which is equal to the difference between the PCE for optimized and non-optimized band gap configurations divided by the “non-optimized” efficiency. Figure 6 depicts this relative efficiency variation as a function of sunlight concentration. Two distinct regions can be observed. The negative region (relative PCE variation <0) represents the range of illumination levels for which a fine-tuning of the electronic gaps does not lead to any improvement in the PCE. The positive region (y > 0), illustrates the illumination range for which a cell design oriented toward resistive losses minimization leads to an increased efficiency.

For low series resistances (Fig. 6), the relative gain in efficiency is quite modest. For instance, a cell optimized for a concentration of 5000 suns shows an improvement in its conversion efficiency (relative to the reference case) at an illumination level of 3700 suns, with a maximum gain of 22%. The relative gain in efficiency for cells optimized for 10000 suns illumination is slightly higher, and the illumination level above which the benefit for bandgap optimization appears is ~5000 suns.

The gain in efficiency is logically more pronounced in the high-*R*_*s*_ scenario (0.05 Ω cm^2^) depicted in Fig. 7. Although modest in the case of a 1000 suns-optimized solar cell, the relative improvement in efficiency can be observed at an illumination level as low as 700 suns, with a maximum improvement of around 40%. Both the illumination level above which an improvement in the efficiency can be obtained, and the gain in efficiency potentially achievable, appear to be significant with *R*_*s*_-optimized cell structures designed for high illumination levels: the gain in efficiency for a 2500 suns-optimized cell appears at 1200 suns with a maximum relative improvement of 95% (the relative gain represents up to 140 and 170% respectively in the case of 5000 suns and 10000 suns-optimized cells).

In this work, 3 J cells have been considered. The trends observed still hold for a larger number of junctions in the stack. However, increasing the number of junctions for a given series resistance value will lower the benefit for bandgap optimization, because of the reduced currents associated with MJ cells involving increasing number of subcells.

It should be stressed that these results provide upper bounds for the maximum efficiency attainable without considering the limiting mechanisms which are usually associated with high bandgap materials (such as decreased minority carrier mobilities, or increased series resistance associated with high-bandgap tunnel junctions). A rigorous assessment of any specific cell architecture, which is not in the framework of this study, should necessarily take into account these limiting factors, as well as a detailed description of the cell parameters (doping, materials parameters, thicknesses, contact geometry, tunnel diodes, etc).

## Conclusions and Prospects

Series resistance losses undoubtedly represent one of the most important limiting mechanisms that restrict solar cell efficiency under illumination levels exceeding several hundreds of suns. In this work, we assessed the improvement in the photovoltaic conversion efficiency tailoring MJ solar cells toward lowering the resistive losses at high illumination levels, allowing us to draw two major conclusions: (1) For illumination levels below ~1000 suns - a typical concentration ratio for today’s state of the art CPV systems - considering series resistance losses does not alter the optimal combination of bandgaps (2) Conversely, we demonstrated a strong improvement in the cell efficiency designed toward minimizing *R*_*s*_-losses for illumination levels exceeding 1000 suns. Increasing the conversion efficiency under ultra-high illumination can thus be achieved through an appropriate bandgap engineering favoring high electronic gaps: despite their lower 1-sun efficiency, such cell architectures allow a significant mitigation of the series resistance losses under high illuminations levels, through a boost in the open-circuit voltage at the expense of the short-circuit current. However, the benefit for bandgap optimization is highly dependent on the ability of other approaches commonly used (such as front grid and emitter optimization) to lower the cell resistance: this strategy offers an extra-degree of freedom in the quest of ultra-low series resistance solar cells, a mandatory condition to be fulfilled to achieve high sunlight to electricity conversion efficiency under very high illumination levels.

We believe that the efficiency paradigm will push the development of solar cells towards accepting ever-higher concentration levels ≫1000 suns). In this goal, series-resistance minimization is of uttermost importance in the cell design, and we show that an appropriate fine-tuning of the electronic gaps in the MJ stack may reduce dramatically the detrimental effect of series resistance losses at ultra-high illumination levels. Another argument for such a development is provided by the analysis of PCE temperature coefficients for CPV cells. It was predicted theoretically^22^ and confirmed experimentally^23^ that absolute values of negative temperature coefficient of CPV efficiency decreases with increasing solar concentration. Efficient operation under concentration of 2000 suns and above may indeed allow CPV cells to operate at elevated temperatures^24^. This, in turn, may open a new research route for development of hybrid PV – “thermosolar”, concentrated solar power (CSP) system^24, 25^ ability.
